# Development and evaluation of an individual innovation model for nursing students: a mixed-method study

**DOI:** 10.1186/s12909-025-08508-6

**Published:** 2026-01-26

**Authors:** Naval Heydari, Mahnaz Rakhshan, Camellia Torabizadeh, Ghasem Salimi

**Affiliations:** 1 Department of Nursing, Community Based Psychiatric Care Research Center, School of Nursing and Midwifery, Shiraz University of Medical Sciences, Shiraz, Iran; 2 Department of Nursing, School of Nursing and Midwifery, Shiraz University of Medical Sciences, Shiraz, Iran; 3 Department of Educational Administration and Planning, Faculty of Education and Psychology, Shiraz University, Shiraz, Iran

**Keywords:** Individual innovation, Nursing, Student, Model

## Abstract

**Background:**

Innovation is a necessary competence in nursing students and exploration of this concept in the context of nursing students is crucial. One of the robust approaches to exploring concepts is model development. So, the aim of this study was development and evaluation of an individual innovation model for nursing students, in order to provide educators with a practical framework for foster innovative skills of nursing students, ultimately improving quality of care in clinical settings.

**Methods:**

This Sequential exploratory mixed-method study was conducted in a nursing school located in the south of Iran in two phases: (1) exploring the concept of individual innovation and its dimensions and (2) psychometric evaluation of the model. In the first phase, the researchers conducted a qualitative research based on content analysis, a scoping review, and model development. In the second phase of the study, the model was evaluated using the Delphi method and the model items were prioritized and weighted using the analytic hierarchy process.

**Results:**

Based on the findings from the first phase of the study, the components of the initial version of nursing students’ individual innovation model consisted of 44 items which fell into three categories: dynamism and comprehensive reform, professional innovation, and alignment of innovation drivers. After being analyzed and revised in the stage of validation, the items decreased to 27.

**Conclusion:**

Individual innovation is a developing, systematic, and multi-faceted phenomenon influenced by a variety of personal, professional, and organizational factors. Management of the influential factors in individual innovation by managers and development of effective interventions in this area can help promote individual innovation among nursing students.

**Clinical trial number:**

not applicable.

**Supplementary Information:**

The online version contains supplementary material available at 10.1186/s12909-025-08508-6.

## Introduction

Individual innovation is encompassing both the willingness to search for and find and apply new approaches to problem solving and the ability to accept, apply, tolerate, and experience new topics [1, 2].Nurses are faced with many challenges in caring. By using innovation, nurses can benefit from novel techniques and technologies more effectively [3]. The International Council of Nurses (ICN) recognized innovation as an essential professional skills for nurses [4].

According to a few studies, to enjoy the benefits of innovation, nurses should be trained in innovation from the beginning of their education [5, 6]. Promotion of innovation is essential to preparing nursing students for using their knowledge, critical thinking skills, and initiative to identify their educational needs and make appropriate responses to patients’ care needs [7, 8]. Thus, innovation is a necessary competence in nursing students [1, 9]. The consequences of fostering this competency are evidenced in researches: A study showed that nursing students enrollment in courses on innovative thinking improved levels of curiosity and exploration [10],another study showed an innovation process in nursing course improve students’ creative thinking tendencies and entrepreneurship skills [11] another study determined that the level of innovative behaviors is associated with professional identity and the frequence of reading scientific literature in nursing postgraduates [12]. , also innovation can affect perception of care in nursing students [5]. Furthermore, studies link nurse-led innovation to tangible outcomes such engineered new smartphone applications with the objectives of guiding healthcare personnel, improving patient access to care, enabling individuals to monitor their health status, and providing support to patients with chronic illnesses [13].

In previous studies there is two qualitative Study in this field: the concept analysis study [6] and a content analysis [14] that have addressed the nature of the concept of innovation in nursing and nursing students, respectively. Most studies conducted in this field are descriptive and cross-sectional and have examined the level of innovation in nurses and nursing students or have examined the relationship of innovation with other variables such as demographic characteristics, leadership style, perception of individualized care, creativity, and use of technology [1, 5, 9, 15, 16], So despite the significant role of innovation in the personal and professional advancement of nursing students, there is a big gap between rhetoric and reality in nurturing innovation in nursing students. There are two critical and interconnected problems according to the current literature. Firstly, although there are known attributes of innovation in general, its meaning, behaviors, and manifestations as an individual characteristic within the uniqueness of the socio-cultural and clinical context of nursing students remain understudied and vague. Second, as a direct result of this conceptual ambiguity is the absence of a valid theoretical model that guides nursing educators. Not having such a model is the primary obstacle to systemically improving innovation in nursing students. Therefore, A study is urgently needed to explore the detailed idea of individual innovation among nursing students. It should also turn that understanding into a practical, validated model. To meet this complex need, a mixed-methods research design is necessary. A purely qualitative approach would be great for initial exploration, but it would not be enough to test the generalizability and structural relationships of the new model. On the other hand, a purely quantitative approach would be too early, since there is no validated model.

Therefore, This study was designed to address a significant gap by creating and validating a model of individual innovation specifically for nursing students. The research unfolded in two distinct, sequential phases: First, a qualitative phase aimed to delve into the experiences, perspectives, and insights of both nursing students, helping to outline the essential elements of the concept.

Next, a quantitative phase was carried out to validate the model derived from the qualitative insights, clarifying the connections between its various components.

By utilizing this thorough mixed-methods approach, the study offers a well-rounded understanding of individual innovation among nursing students, leading to a validated model that can effectively guide curriculum development, teaching methods, and future research focused on nurturing this vital competency.

## Materials and methods

### Study design

**Fig. 1:**
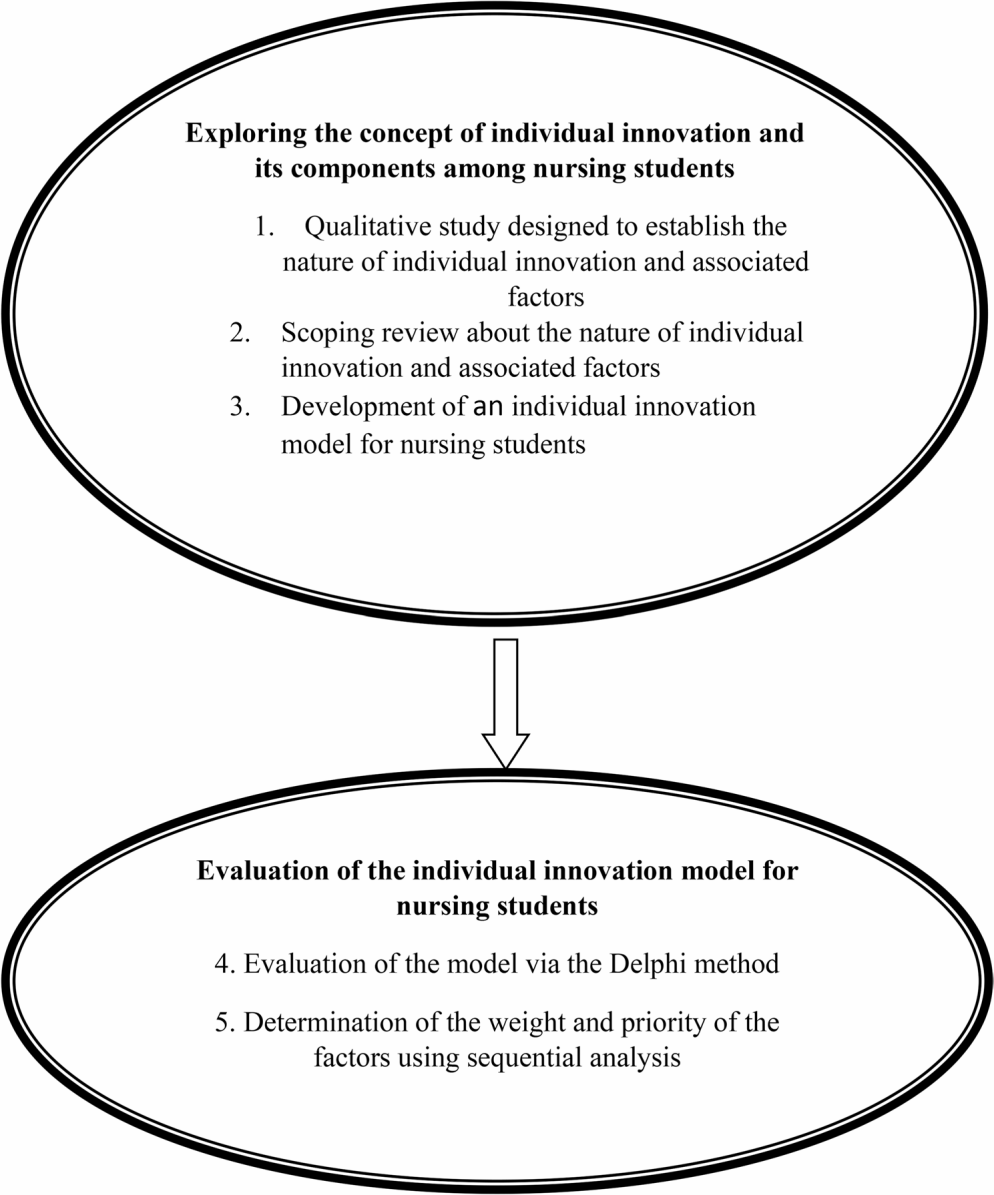
The stages of exploring individual innovation among nursing students and developing a model for it

The present study is a mixed-method work of research with a sequential exploratory design, conducted between September 2021 and January 2024. The study was carried out in two stages: exploring the concept of individual innovation and its components and validating the resulting model. The first stage consisted of three phases: a qualitative study with a content analysis design, a scoping review designed to explore the concept of individual innovation among nursing students, and development of a model with the help of a panel of experts. In the second stage, the model was evaluated using the Delphi method and the model items were prioritized and weighted using the analytic hierarchy process (Fig. 1).

### Participants and sampling

The study population was the nursing students at Shiraz University of Medical Sciences in southern Iran. As the study was conducted in several stages, the manner of sampling in each stage is explained as follows:

#### Recruitment of participants for the first stage (the qualitative phase)

In this stage, 11 nursing students from Shiraz University of Medical Sciences were selected by purposeful sampling. The inclusion criteria were as follow:

- Being a student in a nursing program (bachelor’s degree, master’s degree, or PhD)
- Having a history of developing an innovative work or patenting an invention or being a member of the Society of the Talented and the Accomplished or the National Elites Foundation
- Obtaining a score of above 68 on the Individual Innovativeness Scale developed by Hurt et al. (1977)(17, 18)
- Being willing to participate in the study

The participants who decided to withdraw from the study at the time of or after the interviews or refused to answer the interview questions were excluded.

One of the criteria for being interviewed was obtaining the minimum score on the 20-item Individual Innovativeness Scale developed by Hurt et al. (1977) [17].(supplementary file1) In the present study, the scale in question was back-translated and the Persian version was evaluated. This scale was also evaluated in 2010 using a population of nursing students [18] and nurses [3] in Turkey. The Individual Innovativeness Scale is one-dimensional and consists of 12 positive and 8 negative items, scored on a 5-point Likert scale. The score range is between 14 and 94, and a score of above 68 indicates satisfactory innovativeness [17]. In the study of Turkish nurses, the CVI of the scale was found to be 0.91 [3], and other studies reported the discriminant validity of the scale to be satisfactory [19]. In studies conducted in Turkey, validation of the scale with nursing students resulted in four subscales [18], while validation of the scale with a population of nurses resulted in three subscales [3]. The factor loadings of the items of the original scale were between 0.52 and 0.76 [17]; other studies found them to range between 0.32 and 0.82 [19], 0.36 and 0.78 [18], and 0.49 and 0.75 [3]. The Cronbach’s alpha of the scale equal 0.89, and other studies found it to be 0.80 [19] and 0.82 [3, 18]. The split-half reliability of the scale was 0.92 [17]. In other studies, the test-retest reliability of the scale was (*r* = 0.87, *p* < 0.05) [18] and (*r* = 0.60, *p* = 0.000) [3]. In the present study, the impact score, CVR, and CVI of the scale were found to be above 1.5, between 0.6 and 1, and between 0.8 and 1 respectively. SCVI-Average equaled 0.91. Exploratory factor analysis resulted in three subscales, which explained 55.49% of the variance of the items. The Cronbach’s alpha and intraclass correlation coefficient of the scale were 0.88 and 0.94 respectively [20].

#### Recruitment of participants for the third stage (model development)

The model was developed using feedback from a 20-member panel of experts. The inclusion criteria for the experts were as follows: having a master’s degree or more, having adequate knowledge and experience of management or research in education or nursing, and being willing to participate in the study.

#### Recruitment of participants for the fourth stage (evaluation via the Delphi method)

Use of the classic version of Delphi and heterogeneity in the panel of experts compelled the researchers to recruit 20 experts (some of whom were also on the model development team) by purposeful sampling to evaluate the model. The inclusion and exclusion criteria were the same as in the previous stage.

#### Recruitment of participants for the fifth stage (prioritizing and weighting the model items)

Saaty (2000) recommends 10 experts for analytic hierarchy process [21]. Thus, in this stage of the study, 12 experts from the previous phase were asked to prioritize and weight the model items.

### Data collection and data analysis

#### Data collection and analysis in stage one (qualitative study)

In this stage of the study, data were collected through in-depth, semi-structured interviews using a topical guideline. The main interview question was: “What is individual innovation in nursing students and its associated factor based on your opinion?” The interview guide, developed by the authors specifically for this study, is available as a supplementary file2. Each interview started with an open question, followed by exploratory follow-up questions based on the participants’ responses. The researchers also used interview improvement techniques to enhance the quality of the interviews [22]. The interviewers tried to set aside their own experiences and beliefs and refrain from imposing their views on the participants and interpreting their responses. At the end of each interview, the collected data were summarized and the respondent was informed whether the interview was over or would continue in another session. Each interview lasted between 25 and 55 min. The recorded interviews were transcribed on the same day as they were conducted. Each interview was analyzed before the next interview was conducted. Data collection continued until the data were repeated and new interviews did not enrich the categories (data saturation). In the present study, the data were saturated after 12 interviews, but two more interviews were conducted to ensure data saturation.

The interviews and data analysis were conducted simultaneously. The data were analyzed using the conventional method of qualitative content analysis. Following Graneheim and Lundman’s method, units of analysis were formed by putting the interview transcripts next to each other. Then the transcripts were classified into meaning units, summarized, and labeled by codes. Based on their similarities and differences, the codes were classified into categories and sub-categories. The categories were discussed and revised by the researchers and reflection continued until the researchers unanimously agreed on the codes in each category. Eventually, the underlying meanings were formulated into a theme [23]. After being approved by all the researchers, the resulting codes and categories in this stage were proofread by an expert on Persian literature. The data were analyzed by MAXQDA v. 2018.

To ensure the rigor of the qualitative phase, we considered four criteria suggested by Lincoln and Guba (1985): credibility, dependability, confirmability, and transferability [24]. The researchers’ previous experience in qualitative studies, ongoing involvement with nursing students, triangulation, member checks (which included two PhD students and two undergraduate students), and expert checks of the codes and categories improved the credibility of our study. The participants were selected to provide a range of age and gender, following Hurt and colleagues’ scale of individual innovativeness. Additionally, the research questions were varied to collect enough data. To improve the reliability and credibility of the research, an external observer (a female associate professor of nursing with a PhD in nursing education and 29 years of professional experience) joined the research team during data collection and content analysis. The researchers also explained the study methodology and provided her with the necessary information. We ensured transferability through a detailed description of the categories, participant characteristics, and the methods used for data collection and analysis.

The research in qualitative phase also complied in accordance with tenets of the Helsinki declaration, and has been approved by the Research Ethics Committee of Shiraz University of Medical Sciences. Informed consent was obtained from the participants after clarifying the research objectives and introducing the researchers.

#### Data collection and analysis in stage two (scoping review)

A search in several databases, including PROSPERO and Cochrane library, showed that a systematic review in the domain of exploring individual innovation and its components in nursing students had not been undertaken before. Accordingly, the researchers applied a scoping review to explore the concept of individual innovation and its components in nursing students. Before the review started, the study’s protocol was registered on PROSPERO (registration code: CRD42021260342).

##### Search strategy

The researchers searched the two preferred databases of PubMed/MEDLINE and Embase, as well as the databases of Scopus, Web of Science, Cochrane, ProQuest, and Google Scholar and the Persian databases of Iran Medex, Magiran, Irandoc, and SID.

The keywords were selected according to MeSH, Emtree terms, and synonyms provide on the databases and relevant free words. Studies in the fields of innovation and innovation in nursing were examined to acquire keywords related to the subject under study. Search of keywords in the databases was conducted using the PICOT method (Table 1). The search targeted titles, abstracts, and keywords, and the gray literature was examined too.

**Table 1:** Search strategy for investigating individual innovation and its components in nursing students using PICOT

Strategy	#1 AND #2	PICOT
#1	Students, nursing OR Student, Nursing OR Nurses, Pupil OR Nurse, Pupil OR Pupil Nurses OR Pupil Nurse OR Nursing Student OR Nursing Students	P
	The study does not use an intervention.	I
	The study does not make comparisons.	C
#2	Innovation OR Innovativeness OR Originality	O
#3	Studies conducted in 1962 and after	T

The inclusion criteria for studies were as follows:

1. The qualitative, quantitative, or mixed-method study had to have at least one component of individual innovation in nursing students in its title, abstract, or keywords.
2. Since the oldest study of individual innovation was a study by Rogers (1962) [25] , studies conducted in 1962 and after were included.
3. The articles had to be related to the research question.
4. The article had to come from a journal which uses peer review.
5. Articles submitted to domestic and foreign conventions and valid guidelines and reports released by national and international organizations were also reviewed.

Articles whose full texts were not available due to access limitations were excluded.

##### Screening the search results

Articles were selected by two of the authors using the search keywords. Next, a complete list of the sources of all the selected articles was generated, the titles of the articles were checked by the researchers, and repeated articles and articles not related to the research objective were eliminated. Subsequently, the articles’ abstracts and reports were evaluated by two of the authors and the articles which could contribute to the development of an individual innovation model for nursing students were selected. Since the included articles were cross-sectional studies, the quality of articles were tested by STROBE^1^ [26].

##### Data extraction and results synthesis

The data collected from the reviewed studies were summarized and organized according to setting, design, intervention, outcome, demographics, population, time of study, data collection tools, method, results, limitations and conclusion. Then the collected data were classified and the domains were determined. The extracted data were recorded via Microsoft Word and End note x8.1 was used to manage the resources, save the files of the articles, and eliminate the repeated works.

#### Data collection in stage three (model development)

The general procedure for developing conceptual models in healthcare studies consists of four phases: theoretical interpretation of the main concept, review of the current knowledge and identification of similar models in the domain of the main concept, development of the conceptual model, and validation of the developed conceptual model [27]. In the present study, in three two-hour sessions, 20 experts made suggestions about development of the new model based on the final factors obtained in the previous stages. Next, based on the suggestions of the panel of experts and by using graphic design software programs, including Photoshop and Edraw Mind Pro 9.0.10, the researchers created 13 different versions of the model and presented them to the panel of experts. After discussing the models, the researchers agreed on one of the models, that was conveying the concept and encompassing different dimensions of the model, and also be aesthetically appealing and engaging for the intended audience.

#### Data collection and analysis in stage four (evaluation by the classic Delphi method)

The dimensions and components of the model as extracted from the first phase of the study were converted to a questionnaire and submitted to the panel of experts. The experts were asked to rate the significance of each subscale on a 5-point Likert scale, ranging from very little = 1 to very much = 5. They could also point to items which they regarded as significant but were not included in the questionnaire in the notes section.

Inter-rater agreement was measured using such statistical methods as central indices, agreement, and SPSS v.25. According to previous studies, when subscales are rated on a 5-point Likert scale, the subscales whose mean score is less than 2.5 (50% agreement) should be eliminated, and an agreement rate of 70 to 80% indicates consensus [28]. In the present study, a mean score of 3.5 (70% agreement) was set as the minimum score for retaining a subscale, i.e. for an item to be included in the second round of Delphi, it had to have an agreement rate of 70% or above. Screening should continue until an agreement rate of 80% is achieved. In the present study, consensus was obtained after two rounds of Delphi.

#### Data collection and analysis in stage five (prioritizing and weighting the model items)

The analytic hierarchy process consists of four stages: generation of a decision tree, pairwise comparison, normalization, and determination of priorities and consistency rate [21]. Thus, the research team first determined the factors and criteria of the decision tree (the subscales and components of the individual innovation model for nursing students). Next, a pairwise comparison questionnaire was devised by the research team and 12 experts compared and rated the components of the model two by two on a 9-point Likert scale as suggested by Saaty [21], ranging from equally preferred to extremely preferred. In this stage, the researchers used Expert choice v.11 to analyze the collected data and determine the priorities and consistency rate.

### Validity, reliability, and methodological integrity

In the present study, a combination of qualitative and quantitative data was used to develop the model. In the course of the study, validity, reliability, and methodological integrity were measured through a systematic and precise approach. Trustworthiness and consistency were established by audit trail, regular team management meetings, reflexivity, triangulation and convergent intersection of qualitative and quantitative data.

## Findings

### Findings of stage one (qualitative study)

Overall, 14 interviews were conducted with 11 nursing students from different academic levels. The majority of the participants were female (6), single (6), and aged between 20 and 37 years. The researchers applied maximum variation sampling. The initial 645 codes obtained in this stage were summed up and classified into nine categories and three themes, namely dynamism and comprehensive reform, professional innovation, and alignment of innovation drivers (Table 2).

**Table 2:** Formation of the themes

Theme	Category	Sub-category
Dynamism and comprehensive reform	Personal and professional dynamism	Personal dynamism
		Professional dynamism
	Breaking self-imposed limitations	Avoiding daily routines
		Autonomy in thought and action
	Structural and procedural reform	Reforming the nursing procedures and system
		Adjusting to and accepting reforms
Professional innovation	Brainstorming in nursing	
	Practical manifestations in nursing	
Alignment of innovation drivers	Stimulators of innovation	Deliberation
		Investigating and raising questions
		Deep thinking
		Self-confidence
		Persistence
		Risk taking
		Problem-solving skills
		Clinical decision-making skills
		Acquiring teamwork and inter-disciplinary skills
		Leadership skills
		Updating one’s knowledge
	Internal drivers	Personal interest
		Meeting personal needs
		Believing in innovation in nursing
		Talent for innovation
	External drivers	Motivation and motivators for creativity
		Meeting the needs of profession and society
		Teachable nature of innovation
		Laying environmental groundwork and networks for exploring and creating
	Pro-innovation networks	Support policies
		Support of university
		Support of family and society
		Quality and quantity of access to facilities and financial resources
		Quality of access to individuals and institutes which provide counsel on innovation

#### The theme of dynamism and comprehensive reform

The nursing students regarded dynamism and liveliness in different domains of personal and professional behaviors, trying to go beyond daily routines, creating reform, and adjusting to change as essential to innovation. According to one student:

*“The first thing that comes to mind for me when I hear the word innovation is change and reform; I mean an individual decides to change something to improve a process or a product”* (Participant 1).

#### The theme of professional innovation

From the nursing students’ perspective, innovators always seek new approaches with better potentials in their way of thinking and practice. This theme comprises the two categories of brainstorming in nursing and practical manifestations in nursing.

*“Innovation means having new ideas about exploring uncharted territories in nursing*,* thinking about subjects in nursing science no one has addressed before*,* or thinking about subjects from different and novel perspectives”* (Participant 10).

#### Alignment of innovation drivers

The participants referred to some factors as facilitators in individual innovation among nursing students and described some other factors as barriers to it, and they stressed that the facilitating factors should be aligned for innovation to occur. This theme comprises four categories: stimulators of innovation, internal drivers, external drivers, and pro-innovation networks. Regarding motivation as an internal driver, one of the participants mentioned that:

*“Unfortunately*,* many of the nursing students don’t have much motivation for being innovative in nursing. … One must have a motive for wanting to move toward innovation”* (Participant11).

### Findings of stage two (scoping review)

**Fig. 2:**
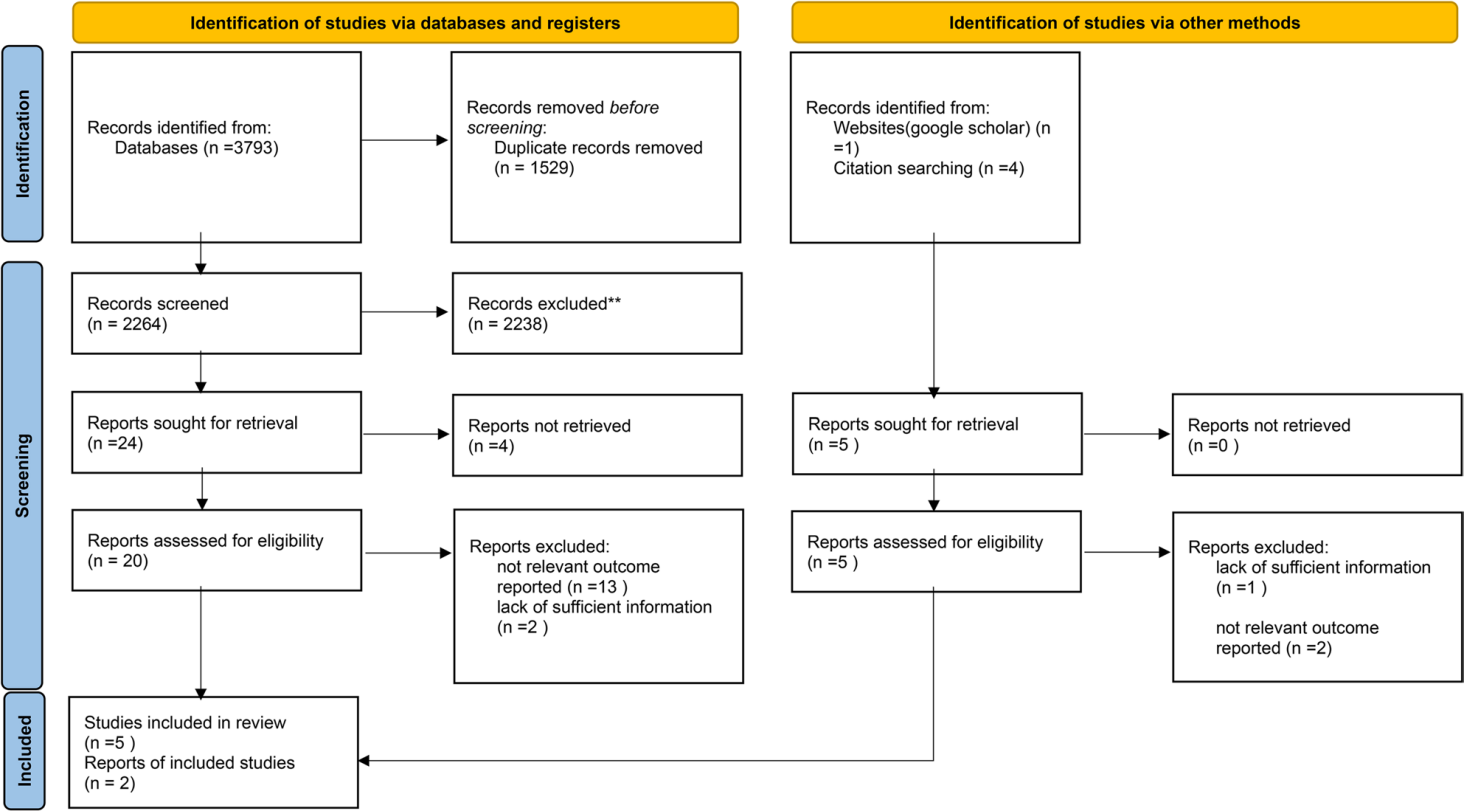
PRISMA 2020 flow diagram From: Page MJ, McKenzie JE, Bossuyt PM, Boutron I, Hoffmann TC, Mulrow CD, et al. The PRISMA 2020 statement: an updated guideline for reporting systematic reviews. BMJ 2021;372:n71. doi: 10.1136/bmj.n71. [29]

In this stage, 3793 articles were extracted, 1529 of which were repeated and thus eliminated. After the titles and abstracts of the remaining 2264 articles were read, 2234 articles were found to be irrelevant to the objective of the study and were thus eliminated. Of the 24 articles which were qualified for full-text review, four articles were eliminated because their full texts were not available. The full texts of the remaining 20 articles were carefully read and five articles which were relevant to the objective of the study were selected. A search of websites, including Google Scholar, and the references of the selected articles, five more articles were included, two of which were relevant to the objective of the study and provided rich information. Review of the gray literature did not yield any articles. Thus, a total of seven studies were found to be aligned with the objective of the study (Fig. 2) .

A review of the selected literature showed the following to be effective factors in individual innovation : education [5, 15], e-health literacy [30], curiosity [9], use of technology in daily life and education, perception of one’s own state of innovation and considering oneself as innovative [1], extent of reading, following the news, and using the Internet and definition of innovation from the students’ perspective [31], organizational obstacles [32], proficiency in a foreign language [15, 32], educational barriers, support of an educational institute and follow-up on innovation [15]. (Table 3) .

**Table 3:** Results of studies that are included in scoping review

factors affecting individual innovation in nursing students	Results	Study design	Study time	Sample size	Corresponding author/ title	num
- Positive Relationship between Individual Innovation and Caring Perception - Education one of the Factors Affecting Individual Innovation	The innovation score of most students was in the early majority category (between 57 and 68). There was a positive correlation between the score of the acceptance of experience dimension of the individual innovation questionnaire and the perception of personal care, and with increasing personal innovation, the perception of care also increased.	Cross-sectional	2018–2019	230	Demirel/ Relationship between individualized care perception and innovativeness among final-year nursing students(5)	1
- E-health literacy is one of the factors affecting individual innovation.	55.9% of students were backward and traditional in terms of individual innovation. There was a significant inverse relationship between e-health literacy score and personal innovation.	Cross-sectional	2018	227	Karadag Arli / E-Health Literacy and Individual Innovation in University Students Enrolled in Health-related Departments(29)	2
- Curiosity about factors affecting individual innovation	The innovation score was quite high. Innovation had a significant correlation with curiosity.	Cross-sectional	2019	98	Liu/ The association between creativity, creative components of personality, and innovation among Taiwanese nursing students(9)	3
-Using technology in life and education, -A person’s understanding of their innovation status and whether they consider themselves innovative	The average individual innovation score was 61.02 ± 8.89 and was placed in the questioning category. Innovation had a positive relationship with the score of the dimensions of the role of technology in life and educational use of technology, but not with the overall score of the technological equipment questionnaire. Individual perception of innovation was also one of the factors affecting innovation.	Cross-sectional	2015–2016	165	Durgun/ Nursing Students’ Technological Equipment Usage and Individual Innovation Levels(1)	4
-Amount of study, -Following the news, -Using the Internet, -Definition of innovation from the perspective of students	The average score of individual innovation was 63.71 ± 10.18. The majority of students were in the questioning category and agreed to use innovation-based courses and tools in education. The amount of reading, following newspapers and magazines, the amount and purpose of using the Internet, and the definition of innovation from the students’ perspective (synonymizing innovation with newness, or creativity, or initiative and invention) had a significant relationship with individual innovation. It was found that there was a significant difference between the levels of individual innovation of students and the perception that the efforts of professional organizations in developing innovative applications are important.	Cross-sectional	2016–2017	590	Reyhan/ Opinions of Midwifery and Nursing Students about the Level of Individual Innovativeness and Innovation in Education(30)	5
-Organizational barriers, -Lack of foreign language proficiency	The average innovation score of students was 63.92 ± 10.06 and was at a low level, and most students were in the early majority category, and the level of innovation of women was higher than that of men. The most important obstacle to innovation was the lack of effective access to resources due to unfamiliarity with a foreign language, and most of the obstacles to innovation were in the organizational category, and the least obstacle was family structure.	Cross-sectional	2015–2016	277	ERTUĞ/ Investigating the Individual Innovativeness Profiles and Barriers to Innovativeness in Undergraduate Nursing Students(31)	6
- Educational barriers, - Innovation training, - Pursuit of innovations, - Foreign language proficiency, - Educational institution support	The average score of students was 63.53 ± 8.5 and was at a low level. The most important barriers to innovation were the lack of various courses to support creativity, the lack of planning appropriate activities to support innovation by educational institutions, and the inability to access current information due to lack of knowledge of a foreign language.	Cross-sectional	2018	279	Ceylan /Innovativeness Levels and Perceived Barriers to Innovativeness of Nursing Students(15)	7

### Findings of stage three (model development)

The model development team consisted of 20 experts aged between 33 and 60 years and with work experience of 2 to 35 years. In this stage, the factors of the individual innovation model for nursing students as obtained in the previous stages of the study were examined and revised by the development team. In the end, the 44 items were organized into the three themes of dynamism and comprehensive reform, professional innovation, and alignment of innovation drivers.

### Findings of stage four (evaluation by the Delphi method)

In this stage, the model was evaluated by 20 experts aged between 31 and 61 years and with work experience of 1 to 35 years. In the first round of Delphi, the inter-rater agreement for all the items was above 70% (mean score = 3.5) and none of the items was eliminated. However, the experts made a few suggestions to enhance the clarity and simplicity of the items and merge similar items, reducing the number of items from 44 to 31. In the second round of Delphi, the categories and items were assessed by the experts again. At this point, inter-rater agreement was above 80% and thus none of the items was eliminated. However, the experts suggested that some overlapping items be merged, reducing the number of items on the model to 27. In view of the high rate of inter-rater agreement in the second round, in the third round, the items were evaluated by only three experts for final approval. The lowest and highest inter-rater agreement mean scores in this stage were 4.33 and 5 respectively.

### Findings of stage five (weighting and prioritizing the themes and items of the model)

A total of 12 experts aged between 31 and 50 years and with work experience of 2 to 25 years participated in this stage. The results showed the theme of dynamism and comprehensive reform to have the highest priority, followed by professional innovation and alignment of innovation drivers (Table 4). The inconsistency ratio of all the items was measured and found to be less than 0.1.

**Table 4:** Prioritizing and weighting the themes and items of the individual innovation model for nursing students

Theme	Weight of theme	Priority	Item	Weight of item	Priority
Dynamism and comprehensive reform	0.453	1	Setting goals and avoiding daily routines	0.264	1
			Autonomy in thought and action	0.253	2
			Personal and professional dynamism	0.198	3
			Reforming the nursing procedures and system	0.187	4
			Adjusting to and accepting reforms	0.098	5
Professional innovation	0.327	2	Innovativeness in nursing	0.537	1
			Originality and creating new ideas in nursing	0.463	2
Alignment of innovation drivers	0.220	3	Stimulators of innovation	0.332	1
			Inclination to take risks and face challenges	0.140	1
			Knowledge and utilization of new technologies	0.114	2
			Problem-solving skills	0.112	3
			Persistence	0.107	4
			Teamwork skills and inter-disciplinary cooperation	0.093	5
			Self-confidence	0.092	6
			Clinical decision-making skills	0.091	7
			Effective leadership	0.073	8
			Investigating and asking questions	0.072	9
			Development of e-health literacy	0.060	10
			Contemplation	0.047	11
			Internal drivers	0.278	2
			Interest in one’s profession and innovation	0.252	1
			Tendency to learn innovation	0.226	2
			Intelligence and talent for innovation	0.219	3
			Believing in innovation in nursing	0.167	4
			Following innovations in nursing	0.136	5
			Pro-innovation networks	0.209	3
			Access to support policies	0.620	1
			Access to support of educational institutes	0.380	2
			External drivers	0.182	4
			Innovation-friendly environment	0.553	1
			Meeting personal, professional, and social needs	0.447	2

**Fig. 3:**
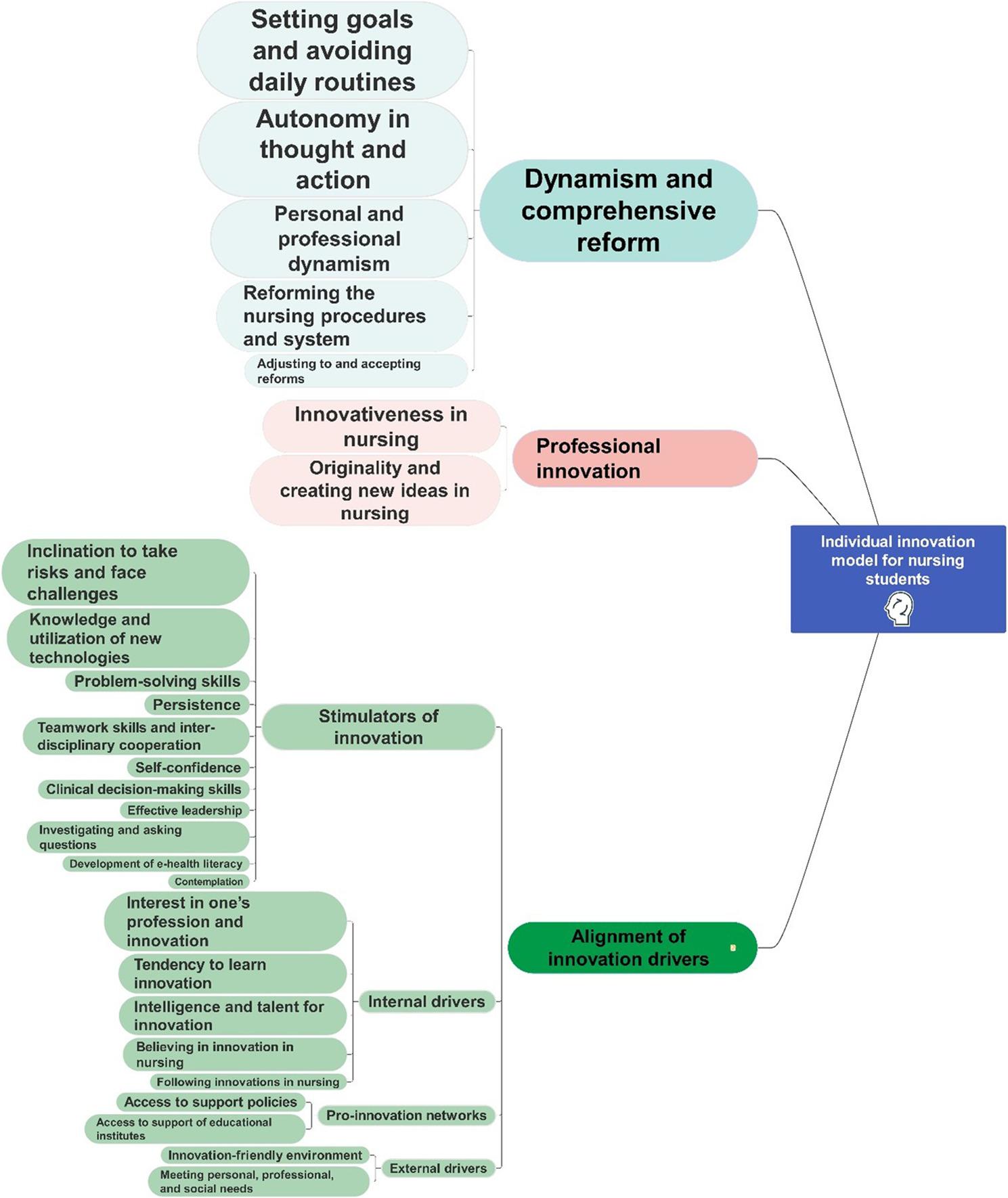
The individual innovation model for nursing students

Eventually, based on the findings of this stage and the weights and priorities of the themes and factors, the final version of the individual innovation model for nursing students was developed (Fig. 3).

## Discussion

The present study developed and evaluated a model for individual innovation in nursing students using a mixed-method approach. The model conceptualize individual innovation not just as a fixed personal trait, but as a dynamic result of three main dimensions: [1] Dynamism and Comprehensive Reform [2], Professional Innovation, and [3] the Alignment of Innovation Drivers. Importantly, these dimensions interact with one another. As shown in Fig. 3,: the internal and external drivers (Dimension 3) energize the student’s dynamism (Dimension 1), which is their desire to challenge routines and seek change. This dynamism is necessary to turn innovative potential into real Professional Innovation (Dimension 2) in practice, education, research, or management. The validated model and its connections offer a solid framework for understanding and promoting innovation in nursing education.

### Dynamism and comprehensive reform

The highest priority theme of the model was dynamism and comprehensive reform, The findings of the present study indicated that individual innovation demands going beyond routines and trying new ways. Similarly, other studies report that repetition and routines hamper innovation as they incline individuals to solve problems with old, familiar solutions and not to seek new approaches [33].

A critical component of dynamism and comprehensive reform is autonomy in thought and action. The participants described disregard for nursing students’ autonomy as an obstacle to individual innovation. The quantitative findings from the model evaluation phase significantly corroborated the importance of this component, with ‘autonomy in thought and action’ receiving one of the highest weightings among the sub-components of the dynamism theme. This convergence between the qualitative data (which identified a lack of autonomy as a barrier) and the quantitative data (which quantified it as a key factor) underscores the indispensable role of autonomy as a prerequisite for innovation. According to previous studies, allowing nurses to act independently encourages them to be innovative. Thus, autonomy and independence are essential to promotion of individual innovation [34, 35].

Personal and professional dynamism is another dimension of dynamism and comprehensive reform. Professional dynamism has not been addressed in many studies. In the present study, however, in addition to personal dynamism, the participants stressed the role of dynamism in all the domains of nursing education, research, management, and clinical practice for individual innovation to happen. Dynamism and action are among the tools which enable care providers to generate new ideas. Maintaining dynamism in thinking results in innovative and viable ideas which can improve the healthcare system [36]. A systematic review confirmed that dynamism contributes to innovative practices [37]. Likewise, Mohammadi Golafshani (2017) found a significant positive correlation between environmental dynamism and innovation [38].

Another aspect of innovation is reforming the nursing procedures and system. In some sources, innovation is regarded as synonymous with reform [17]. Some other sources define innovation as adjustment to change in providing services and presenting ideas and new ways of performing one’s tasks [39]. In a study by Sahsavar Isfahani et al. (2015), having an open mind about change is one of the characteristics of creative clinical nurses [33]. According to a cross-sectional study of operating room nurses in Turkey, nurses with higher levels of individual innovation were able to adapt to new methods, e.g. robotic surgery, in shorter periods [40].

### Professional innovation

Another dimension of the model is professional innovation, which involves innovative performance and generating new ideas in nursing. In a study conducted to explore the concept of innovation in nursing, “thinking creatively” was one of the themes extracted from the interviews with nurses [41], which is similar to the subscale of “creating new ideas in nursing” on the present model. In the present study, the participants also referred to innovative acts in nursing practice, management, education, and research. Similarly, other studies verify that innovation involves generating new ideas, using existing ideas in novel ways, or trying new approaches intended to utilize new ideas in practice [41, 42]. An innovative person is one who is not only able to come up with new ideas, but can transform ideas into innovative practices [43]. Thus, originality is the gateway to innovation in nursing.

### Alignment of innovation drivers

The theme of alignment of innovation drivers consists of four categories: stimulators of innovation, internal drivers, pro-innovation networks, and external drivers. The participants listed the personal qualities ( e.g., willingness to take risks and face challenges, knowledge and application of new technologies, problem-solving skills) as drivers of individual innovation in nursing students. The findings of the present study provide a more comprehensive insight into the personal characteristics which influence individual innovation than other studies. Previous research confirms the correlation between personal characteristics and creativity and innovation. With regard to creativity, the cognitive and psychological state of an individual mediates the underlying factors in occurrence of creativity in him/her [33, 43]. Formation of individual innovation depends on personal capabilities consistent with Csikzentmihalyi’s systems model of creativity (1999). Csikzentmihalyi regards personal capabilities as influential factors in occurrence of creativity and states that cognitive capabilities are essential to creating new ideas. He names perseverance, having an open mind about experience, and seemingly contradictory behaviors as essential to creativity and innovation [44]. According to a review study by Dedehayir et al. (2017), innovation is influenced by such personal characteristics as concentration, familiarity with technology, knowledge, economic values, up-to-dateness, idea management, and communication [45]. Thus, it can be inferred that individual innovation is a function of personal capabilities and characteristics [43] and that psychological empowerment of individuals can encourage them to be creative [46]. A few studies have even found that personality traits are more effective than physical and organizational factors in motivating the personnel in healthcare centers to be creative and innovative, and personal qualities play an important role in understanding problems and synthesizing new data [9]. Therefore, the decision-makers in nurse education should take measures to develop capabilities which stimulate individual innovation in nursing students.

Other drivers of individual innovation in nursing students are internal and external drivers. According to a study by Fischer at al. (2019), intrinsic and extrinsic motivators have a significant impact on innovation [47]. In another study conducted to explore the creativity process in clinical nurses, intrinsic and extrinsic motivators were found to be effective factors in creativity [48]. Similarly, the results of a study by Evli (2025)showed that it is essential to support students’ professional self-concept as well as their intrinsic and extrinsic motivation to foster innovative behaviors among nursing students [49].

In the model developed in the present study, the internal drivers had a higher weight and priority than the external drivers, and motivation and interest in one’s profession and innovation were at the top of the list of internal drivers. A study of intern nursing students also found a positive correlation between motivation and innovativeness [50]. According to a systematic review, intrinsic motivators, including interest, have a greater impact on innovation than extrinsic motivators [51] because they create satisfaction, encourage individuals to take on complicated, challenging tasks, and increase energy for innovativeness [48]. Accordingly, to foster innovation in nursing students, nurse education systems should seek ways to enhance intrinsic motivators of individual innovation in this population [52]. This conclusion that intrinsic motivators are more potent offers a critical insight for nursing educators: rather than relying solely on external incentives like grades or rewards, efforts should focus on nurturing students’ intrinsic love for learning and innate curiosity.

Among the extrinsic drivers, an innovation-friendly environment had the highest weight in promoting innovation. The environment’s profound role is well-documented [34, 43, 53]. The important role of environment is highlighted by the fact that early exposure to innovation fostering lifelong innovativeness [54]. supportive work environments can improve nurses’ mental health and incline them to act innovatively . Therefore, nurse managers are recommended to use policies designed to create a suitable environment for promoting innovation [34, 35].

Finally, Pro-innovation networks, concern access to support policies and support of educational centers is crucial. According to Ceylan (2019), one of the obstacles to innovation is educational organizations’ failure to develop plans which encourage innovation [15]. One of the strategies for promoting innovation in nursing schools is supporting innovative students and teachers: supporting innovators raises interest in innovation and increases participation in the innovation process [15, 55]. Innovative ideas may encounter obstacles in and outside the organization; thus, to prevent individuals and teams from losing interest in developing innovative plans, they should be provided with adequate support [56].

### Synthesizing the dimensions

Based on the validated model, the three dimensions create a dynamic, interdependent system instead of separate factors. The alignment of innovation drivers, which includes internal motivators, external supports, and pro-innovation networks, is the crucial fuel that activates dynamism and comprehensive reform. This fuel empowers students with the autonomy, energy, and reform-oriented mindset to move beyond routine. This dynamism is a necessary behavioral precursor that leads to the concrete result of professional innovation. This result involves generating and implementing new ideas in practice, education, research, or management. Successful innovation creates a positive feedback loop: it boosts internal drivers like confidence and motivation, which helps maintain the cycle of dynamism and further innovation. Therefore, the model views individual innovation as a continuous, synergistic process. Aligned drivers allow for dynamic action, producing innovative outcomes that then re-energize the entire system.

### A comparison between the innovation model for nursing students and the instructional model development to enhance the ability to create nursing innovation

A review of literature showed that only one study had presented a model for promoting innovation in nursing. The “instructional model development to enhance the ability to create nursing innovation,” developed by Ekthamasuth et al. (2022) in Thailand, was created based on design thinking and reflective practice. This instructional model comprises five stages: (1) preparation and inspiration, (2) data discovery and problem identification, (3) information retrieval and verification solutions, (4) development and inspection of innovation prototypes, and (5) dissemination and reflection on learning [57]. A difference between the instructional model and the model developed in the present study is that the former described the process in which innovation occurs, while the present study explored the concept of individual innovation, defined it, and identified the influential factors in individual innovation. Both studies were designed to find ways of promoting innovation in nursing students, but they used different methods: the Thai study only applied the qualitative approach of content analysis and focused on design thinking, while the present study used a mixed-method approach to explore the concept of individual innovation from different angles, including a review of literature in addition to qualitative research, and obtained richer and more comprehensive data. Moreover, in Ekthamasuth’s study, only senior nursing students were interviewed, but in the present study, the participants were nursing students from different academic years and levels. With regard to the subscales of the two models, the phase of preparation and inspiration in the instructional models is similar to the subcategory of originality and generating new ideas in nursing under professional innovation in the present model. The phase of data discovery and problem identification resembles the subcategories of investigating and raising questions and problem-solving skills under stimulators of innovation.

### Limitations and suggestions for future research

The setting of the present study was only one university, the use of a purposive sample from a single university in Iran limits the generalizability of the findings. The model should be validated with more diverse populations of nursing students across different cultural and educational settings to assess its broader applicability. Therefore, it is suggested that future studies be conducted on national and international levels and use other methods of research to enhance the body of knowledge in this area and investigate other influential factors in nursing students’ individual innovation. Moreover, in view of the great significance of team innovation in higher education, it is suggested that this concept should be explored in the context of nurse education.

## Conclusion

Based on the findings of the present study, individual innovation in nursing students depends on dynamism and comprehensive reform, coupled with professional innovation and alignment of innovation drivers. The developed model illustrates the concept of individual innovation in nursing students and provides a better understanding of the factors which influence it, and can be used to promote individual innovation in nursing students. The findings of the study showed that individual innovation is a developing and systematic phenomenon, which is affected by a variety of personal, professional, and organizational factors. Therefore, mediating the influential factors in individual innovation and development of effective policies and interventions using this model can contribute to the promotion of individual innovation among nursing students, which should be a part of the official education and professional socialization of nursing students.

## Supplementary Information

Supplementary Material 1.

Supplementary Material 2.

## Data Availability

The datasets used and/or analysed during the current study are available from the corresponding author on reasonable request.

## Footnotes

1 - Strengthening the Reporting of observational studies in epidemiology.
