# Supplementary material for: Development and evaluation of an individual innovation model for nursing students: a mixed-method study
Source: BMC Med Educ. 2026 Jan 26;26:321. doi: 10.1186/s12909-025-08508-6 (PMC12918588; doi:10.1186/s12909-025-08508-6)
Supplement: Supplementary file 1 — Supplementary Material 1. [file 12909_2025_8508_MOESM1_ESM.docx]

**Interview Guide – Developed by the Authors**

**Title of study:** Development and evaluation of an individual innovation model for nursing students: a mixed-method study

**Language Used in Interviews**: Persian

**Interview Format**: Semi-structured

**Introduction**

This interview guide was designed and developed entirely by the authors to support the objectives of a mixed-methods research study. It was used throughout the qualitative data collection phase to ensure consistency, depth, and relevance in participant responses. The guide was tailored to explore specific themes aligned with the study’s conceptual framework.

Section I: Demographic Questions

1. Age2. Gender3. Educational level 4. history of developing an innovative work or patenting an invention or being a member of the Society of the Talented and the Accomplished or the National Elites Foundation

Section II: Core Interview Questions

These questions were designed to elicit detailed responses regarding participants’ perceptions, experiences, and suggestions:

1. Based on your experience, what does individual innovation in nursing mean? Or what is its concept?
2. Can you describe your typical experience with individual innovation in nursing?
3. In your opinion, what dimensions does individual innovation have?
4. Based on your experience, what factors facilitate and what factors hinder innovative ideas? / What are the key challenges you encounter in this context?
5. In your opinion, how would you improve current practices or conditions to enhancing individual innovation among nursing students?

Section III: Concluding Questions

1. Is there anything else you’d like to share regarding this topic?

Notes on Implementation

Interviews were conducted individually in a private setting.

Follow-up questions and probing techniques were used when needed.

Ethical protocols including informed consent and confidentiality were strictly followed.
