# Supplementary material for: Development and evaluation of an individual innovation model for nursing students: a mixed-method study
Source: BMC Med Educ. 2026 Jan 26;26:321. doi: 10.1186/s12909-025-08508-6 (PMC12918588; doi:10.1186/s12909-025-08508-6)
Supplement: Supplementary file 2 — Supplementary Material 2. [file 12909_2025_8508_MOESM2_ESM.docx]

Dear respondent,

The present questionnaire assesses the status of your individual innovativeness. The results will be used in educational planning toward developing the profession of nursing. Your participation in the study is on a voluntary basis and your responses will be anonymous and remain confidential. Please determine the extent to which each item is true for you by checking a box in front of it.

Thank you in advance for your cooperation.

| Item | Strongly disagree | Disagree | Neutral | Agree | Strongly agree |
| --- | --- | --- | --- | --- | --- |
| 1. My peers often ask me for advice or information. |  |  |  |  |  |
| 2. I enjoy trying new ideas. |  |  |  |  |  |
| 3. I seek out new ways to do things. |  |  |  |  |  |
| 4. I am generally cautious about accepting new ideas. |  |  |  |  |  |
| 5. I frequently improvise methods for solving a problem when an answer is not apparent. |  |  |  |  |  |
| 6. I am suspicious of new inventions and new ways of thinking. |  |  |  |  |  |
| 7. I rarely trust new ideas until I can see whether the vast majority of people around me accept them. |  |  |  |  |  |
| 8. I feel that I am an influential member of my peer group. |  |  |  |  |  |
| 9. I consider myself to be creative and original in my thinking and behavior. |  |  |  |  |  |
| 10. I am aware that I am usually one of the last people in my group to accept something new. |  |  |  |  |  |
| 11. I am an inventive kind of person. |  |  |  |  |  |
| 12. I enjoy taking part in the leadership responsibilities of the group I belong to. |  |  |  |  |  |
| 13. I am reluctant about adopting new ways of doing things until I see them working for people around me.. |  |  |  |  |  |
| 14. I find it stimulating to be original in my thinking and behavior. |  |  |  |  |  |
| 15. I tend to feel that the old way of living and doing things is the best way. |  |  |  |  |  |
| 16. I am challenged by ambiguities and unsolved problems. |  |  |  |  |  |
| 17. I must see other people using new innovations before I will consider them. |  |  |  |  |  |
| 18. I am receptive to new ideas. |  |  |  |  |  |
| 19. I am challenged by unanswered questions. |  |  |  |  |  |
| 20. I often find myself skeptical of new ideas. |  |  |  |  |  |
